# Human Papilloma Virus Vaccination and Cervical Screening in the Italian Regions: An Overview of the Current State of the Art

**DOI:** 10.3390/vaccines12050504

**Published:** 2024-05-07

**Authors:** Angela Bechini, Claudia Cosma, Giulia Di Pisa, Alice Fanfani, Giulia Ionita, Davide Liedl, Carla Lunetta, Linda Martorella, Silvia Mele, Lorenzo Stacchini, Gabriele Vaccaro, Ornella Zuccaro, Stefano Valente, Gian Marco Prandi, Paolo Bonanni, Sara Boccalini

**Affiliations:** 1 Department of Health Sciences, University of Florence, 50134 Florence, Italy; angela.bechini@unifi.it (A.B.); paolo.bonanni@unifi.it (P.B.); sara.boccalini@unifi.it (S.B.); 2 Medical Specialization School of Hygiene and Preventive Medicine, University of Florence, 50134 Florence, Italy; giulia.dipisa@unifi.it (G.D.P.); alice.fanfani@unifi.it (A.F.); giulia.ionita@unifi.it (G.I.); davide.liedl@unifi.it (D.L.); carla.lunetta@unifi.it (C.L.); linda.martorella@unifi.it (L.M.); silvia.mele@unifi.it (S.M.); lorenzo.stacchini@unifi.it (L.S.); gabriele.vaccaro@unifi.it (G.V.); 3 Department of Prevention and Public Health, Local Health Authority Asl Roma 2, 00182 Rome, Italy; ornella.zuccaro@aslroma2.it; 4 MSD Italia Srl, 00189 Rome, Italy; stefano.valente@msd.com (S.V.); gian.marco.prandi@msd.com (G.M.P.)

**Keywords:** HPV, immunization, primary prevention, secondary prevention, cervical cancer, women, adolescents, recommendation, elimination, Italy

## Abstract

Human Papilloma Virus (HPV) infection and HPV-related cancers can be prevented through vaccinations and mass cervical screening programmes. The Ministry of Health in Italy provides recommendations on primary and secondary prevention of HPV-related diseases, but the 19 Italian regions and 2 autonomous provinces have organisational and decision-making autonomy, with differences in the strategies for offering prevention. The aim of this study is to describe the HPV vaccination and cervical screening offered in all Italian regions. Regional official documents up until 31 December 2021 were first identified. Subsequently, primary and secondary prevention experts from each region were interviewed to validate the previously collected data. The National Immunisation Plan (NIP) 2017–2019 recommends HPV vaccination from the age of 11 for both sexes, with a coverage target of 95%. HPV vaccination is offered free of charge or co-payment. All regions have screening programmes for cervical cancer, using PAP or HPV-DNA tests every three to five years. All regions have an electronic registry for vaccination and screening status. All regions have developed awareness-raising campaigns. It is important to harmonise regional policies with the implementation of information systems integration. The collected data could enhance both regional and national public health initiatives, bolstering the resilience of vaccination programs.

## 1. Introduction

Human Papilloma Virus (HPV) infection, which often progresses asymptomatically, could persist and cause anogenital warts, anogenital cancers (cervix, anus, vulva, vagina and penis), head and neck cancers (oropharynx, larynx and other regions) and recurrent respiratory papillomatosis [1,2].

Over the years, there has been a downward trend in the incidence of cervical cancer cases due to the HPV vaccine and in mortality associated with the same cancer due to cervical screening and treatment advances [3]. However, cervical cancer is the fourth most frequently diagnosed cancer and the second most diagnosed cancer in women after breast cancer and almost all of them are caused by HPV [4]. About 70% of cervical cancers worldwide are caused by HPV types 16 and 18 [5]. Furthermore, cervical cancer is the fourth cause of cancer death in women, with over 600,000 new cases worldwide every year and 342,000 deaths in 2020 [4,6]. It is estimated that during the next 40 years, about 19 million women will die due to cervical cancer without adhesion to screening and preventive treatment [7]. Furthermore, in the USA, the estimated average number of cancers likely caused by any HPV type in men amounts to 15,500. This includes 900 cases of penile cancer, 2100 cases of anal cancer, and 12,500 cases of oropharyngeal cancer [8]. In Europe in 2022, incidence and mortality rates for cervical cancer were, respectively, 11.7 and 5.3 per 100,000, while in Italy, they were 7.4 and 3 per 100,000 [9]. Addressing the burden of HPV-related cancers and diseases and achieving elimination or, at least, control requires a comprehensive approach according to a 5-key-pillars bottom-up strategy that includes screening, vaccination, treatment, infrastructure strengthening, surveillance and monitoring. Italy could be one of the first countries in Europe to eliminate cervical cancer thanks to the strong political willingness to act on the goal of elimination. In 2021, Italy launched the Italian elimination strategy and call to action with the “Manifesto for Cervical Cancer Elimination” endorsed by the most relevant associations and scientific societies in Italy [10].

In recent years in many countries, such as Italy, the quadrivalent vaccine was replaced by a 9-valent vaccine, which offers a wider protection against the HPV types involved in 90% of cervical cancer cases [11]. The tests for cervical cancer screening are the PAP-test and HPV test (HPV-DNA test). The HPV vaccination reduces the risk of persistent HPV 16/18 infection, high-grade cervical intraepithelial neoplasia (CIN2+ or higher) and adenocarcinoma in situ [5]. Concerning the quadrivalent vaccine, long-term follow-up studies (LTFU) in the Nordic regions have shown continuous protection for more than 14 years [12], and concerning the more recent nonavalent vaccine, studies have shown protection for more than 10 years [13]. As for the quadrivalent vaccine, long-term follow-up (LTFU) studies in the Nordic regions have demonstrated continuous protection through a more than 14-year follow-up [12,13]. Strengthening of vaccination programs is a central point of public health [14]. The World Health Organization (WHO) and the European Union have launched the challenge of elimination of cervical uterine cancer [15,16]. Achieving 90% HPV vaccination coverage in girls, 70% screening coverage and 90% coverage for treatment of precancerous lesions and invasive cancers can reduce the average cervical cancer incidence rate by 10% by 2030 and prevent 70 million cases and 62 million deaths by 2120 [17]. In fact, in less than ten years of HPV vaccination, countries that achieved high vaccination coverage experienced a 73–85% drop in HPV prevalence and a 41–57% drop in high-grade lesions among young women. According to data, it appears that a similar percentage could defeat cervical cancer as a public health problem within a century at least in most lower–middle-income countries (LMIC) [18]. However, the achievement of these results still seems far away globally, considering that currently, only 5 out of 194 nations have exceeded this threshold while 87 have not yet proceeded with the introduction of the HPV vaccination [19]. About 47 million women (95% Confidence Interval, CI 39–55 million) received the full course vaccination, representing a total female population coverage of 1.4% (95% CI: 1.1–1.6), and 59 million women (95% CI 48–71 million) had received at least one dose, representing a total population coverage of 1.7% (95% CI 1.4–2.1) [20].

Vaccination coverage is the best indicator to monitor immunisation preventive strategies, as it provides information on their actual improvement and on the efficiency of the vaccination system. Targets are defined internationally by the Global Vaccine Action Plan 2011–2020 [21] and in Italy by the National Prevention Plan 2020–2025 [22]. In Italy, the vaccination schedule provides the administration of two doses at 0 and 6 months for male and female subjects up to 13 or 14 years, or three doses at 0, 1–2 months and 6 months for older age groups. Immunisation against HPV can also be offered to additional cohorts of females with three doses [23]. Italian data regarding HPV vaccination coverage for the primary target (adolescents in their twelfth year of age) provided by the Ministry of Health and updated on 31 December 2021 are not comforting: compared to 2019, there was a drop in vaccination coverage in 2020, also due to the organisational difficulties caused by the health emergency due to the COVID-19 pandemic. In Italy, during 2021 there was a slow recovery both for females and males, but no region or autonomous province reached 95% of vaccination coverage in any cohort, thus not satisfying the objectives of the National Immunization Plan (NIP) 2017–2019 for HPV coverage at the age of 12 years, neither for girls nor for boys [23].

As a matter of fact, according to the last available data, HPV vaccination coverage of the 2009 female birth cohort is 32.2%. The coverage level rises to 53.5% for those born in 2008 and 70.6% for the 2005 female birth cohort. A closer examination of the data for males reveals lower vaccination coverage rates, with rates of 26.8% for those born in 2009 and 44.0% for those born in 2008. Regarding the 2009 cohort, regions with the highest rates of full-cycle vaccinations are the autonomous province of Trento (61.7% for females and 57.0% for males), Tuscany (57.2% for females and 45.4% for males) and Umbria (52.2% for females and 48.9% for males). However, a high variability in coverage rates for the full vaccination cycle is observed in the different Italian regions [24].

Regarding the secondary prevention strategy, in Italy, the PAP-test is offered every three years to women between the ages of 25 and 64 years, and it is the primary test from 25 to 30 years. It is based on sampling cells from the cervix to identify cellular changes. The HPV-DNA test is based on the detection of high-risk HPV genotypes. The test is offered from the 30th year of age every five years in case of a negative result. If the HPV-DNA test is positive, the patient must be tested with a PAP-test as a secondary examination to select women with cellular alterations who must undergo colposcopy. If the cytology shows no alterations, the woman will repeat the HPV-DNA test after one year [25].

National recommendations on primary and secondary prevention of HPV-related diseases were provided by the Italian Ministry of Health, but the 21 Italian regions and autonomous provinces have organizational and decision-making autonomy. Therefore, there are some differences in regional strategies in offering primary and secondary HPV prevention in terms of age cohorts and target populations. Moreover, changes to cervical screening with the switch from the PAP-test to the HPV-DNA test as the primary examination for older women, as well as digital transformations and the implementation of primary and secondary prevention databases and their linkage, are still ongoing.

Due to this situation, the aim of this study is to collect data on the offer of HPV vaccination and cervical screening in all Italian regions and on the different current population targets to whom HPV vaccination and screening are offered free of charge, actively (with invitation letter or other messages sent to the family address) or not actively (in a passive manner), according to regional programmes. Therefore, the main purpose of this study is to provide an overview of the current state-of-the-art of HPV prevention programmes in the Italian regions in order to understand how to support a future resilient and sustainable system.

## 2. Materials and Methods

The research was divided into two phases: a review of official regional recommendations and a survey for the validation of official documents retrieved. The data were collected from June 2021 to September 2022. In the first phase, which took place from June to December 2021, we explored official regional websites, particularly their sections dedicated to public health, and searched documents issued by regions, autonomous provinces and Local Health Authorities (LHA) up to 31 December 2021, combining keywords relating to HPV and its prevention. We also considered additional documents relating to HPV prevention strategies, if they were cited in official documents issued by regions, autonomous provinces and LHA. All documents were critically subjected to a double check by two authors; in case of doubtful interpretation, an independent reviewer was involved. All documents were recorded in a dedicated database.

In the second phase, between June 2022 and September 2022, primary and secondary prevention experts from each region were identified and interviewed in order to validate data collected during the first phase. Our prevention experts were healthcare professionals serving in the region’s health management or prevention departments of the local health authority. An invitation email was sent to at least two primary prevention experts and two secondary prevention experts for each region. If there was no response, we sent a second email. The experts, who made themselves available, were interviewed. All data sources, roles, and affiliations of the expert interviewed were recorded.

For each region, a database was drawn up containing names, affiliations and contacts of the experts that were interviewed, documents and websites that were consulted, and any information that was gathered during the research process, if validated by the experts.

For HPV vaccination regional programs, the following variables were collected: age and gender for vaccine administration; additional cohorts/categories to whom the vaccine is offered beyond those mentioned in the NIP; vaccination charge for additional cohorts; maintenance of free-of-charge for conditions mentioned in the NIP; duration of gratuitousness (if any); whether vaccination is offered actively (by sending an invitation via letter or phone call) or not actively; development of outreach campaign for HPV primary prevention strategy; existence of an electronic register for recording the vaccination status of subjects undergoing the program; integration of the immunisation register with a register of HPV secondary prevention.

The vaccination offer can be “free and active” (active, i.e., on direct call by letter or phone call), “free and inactive” (inactive, i.e., without direct call), in “co-payment” (by payment of a co-payment that covers a smaller portion of the total cost of the vaccine), or by payment of the full cost of the vaccine.

For HPV screening regional programs, the following variables were collected: the existence of one or more public and organised screening programs within the region; the type of test offered (HPV-DNA test, PAP-test or both); age for test execution by type of test; frequency of test execution by type of test and age group; percentage of subjects invited to be screened in the target population by age group; development of outreach campaign for HPV secondary prevention strategy by type; existence of an electronic register for recording the screening status of subjects included in the program; integration of the screening register with the register of HPV primary prevention.

## 3. Results

### 3.1. Collection of Regional Documents and Adherence to the Validation Phase of the Study

In the first phase of the study, documents on regional recommendations for HPV primary and secondary prevention strategies adopted up to December 2021 were retrieved for all the regions. Among the 21 regions, 16 provided answers regarding both the primary and secondary prevention, with a response rate of 76.2%.

Although validation by at least two experts per each area (primary and secondary prevention) was expected, it was not always possible.

At the end of the validation phase, after sending a second invitation email to all non-respondent experts, no response was received from the following regions:

- For primary prevention: Campania, Emilia-Romagna, Sardinia;
- For secondary prevention: Campania, Friuli-Venezia Giulia, Liguria, Sardinia, Sicily.

Consequently, a validation of the data collected in the first phase was not possible for the previous regions.

**Figure 1:**
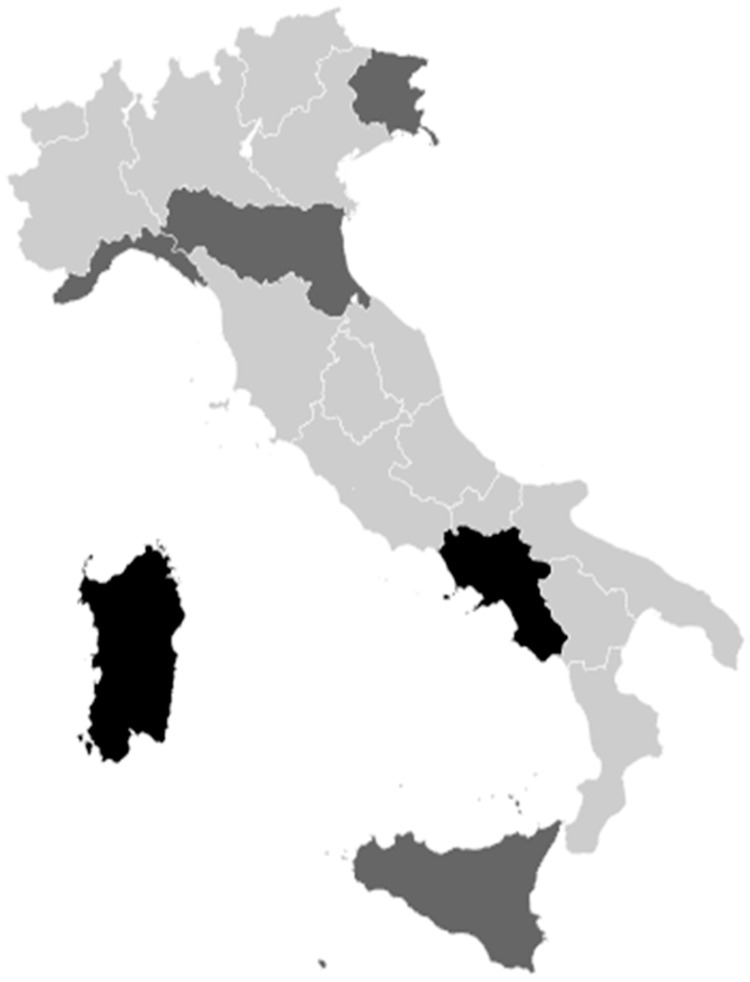
Adherence to the validation phase of the study (light grey: experts validated data for primary prevention and secondary prevention; grey: experts validated data for primary prevention or secondary prevention; black: experts did not validate any data).

An overall view of the adherence to our study is reported in Figure 1.

### 3.2. Primary Prevention Strategy in the Italian Regions

According to the NIP, all the Italian regions have an HPV vaccination offer in the adolescent range of age. For all regions, the offer starts from 11 years, both for females and males.

**Figure 2:**
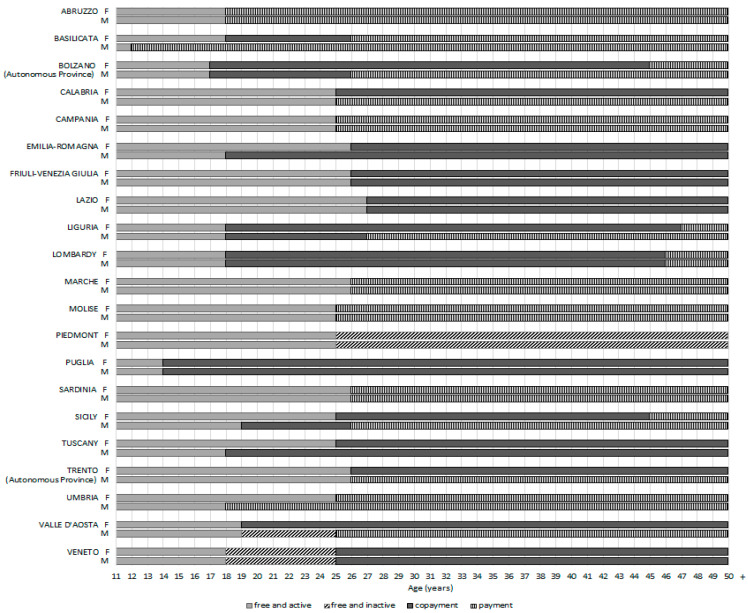
Range of ages of free-of-charge offer and co-payment of HPV vaccines for F (females) and M (males) without risk factors.

The duration of the free-of-charge offer is different in each region: up to 14 years old in Puglia; up to 17 years old in the autonomous province of Bolzano; up to 18 years old in Abruzzo, Basilicata (females), Emilia-Romagna (males), Liguria, Lombardy, Tuscany (males), Umbria (males), and Veneto; up to 19 years old in Sicily (males) and Valle d’Aosta; up to 25 years old in Calabria, Campania, Molise, Sicily (females), Tuscany (females) and Umbria (females); up to 26 years old in the autonomous province of Trento, Sardinia, Emilia-Romagna (females), Friuli-Venezia Giulia and Marche; up to 27 years old in Lazio. In Piedmont, the vaccination is free of charge from the age of 11 years, without any deadline. Depending on the region, the offer is free of charge and actively carried out for cohorts of females born from 1993 to 1997 and males born from 2001 to 2006. For cohorts born before those years, it is possible to receive the vaccination on request in co-payment in most regions (Figure 2).

**Figure 3:**
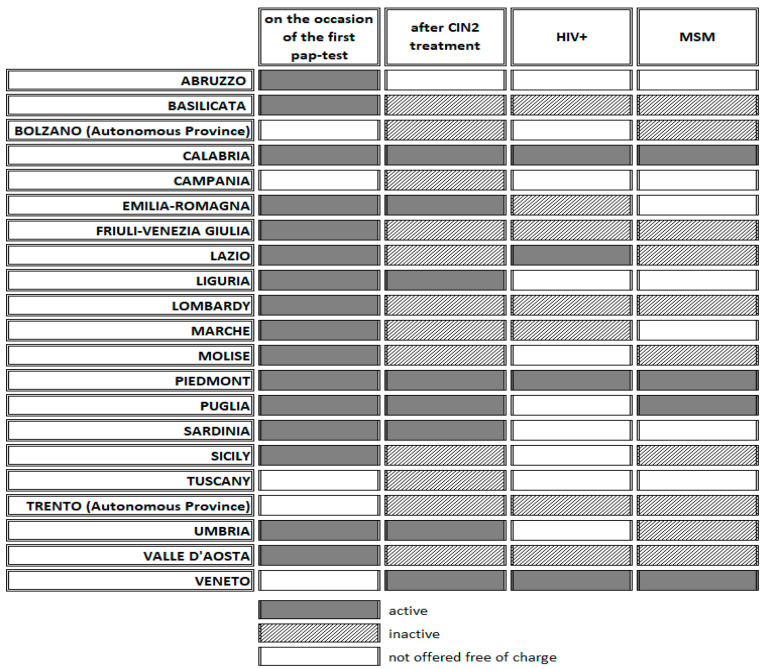
HPV primary prevention for certain subpopulations based on risk factors by region (active or inactive vaccination is free of charge).

In 16 out of 21 regions, vaccination is offered free of charge to women during the first PAP-test screening call. Vaccination is actively carried out in all these regions (Figure 3).

In all regions except Abruzzo, vaccination is offered free of charge on request to women with cervical HPV lesions; moreover, it is actively carried out in Calabria, Emilia-Romagna, Liguria, Piedmont, Puglia, Sardinia, Umbria and Veneto. In Basilicata and Molise, it is also offered free of charge on request to women with a positive HPV-DNA test.

In 11 regions (Basilicata, Calabria, Emilia-Romagna, Friuli-Venezia Giulia, Lazio, Lombardy, Marche, Piedmont, Valle d’Aosta, Veneto and the autonomous province of Trento), vaccination is offered free of charge to HIV positive patients who request it, except for 4 regions (Calabria, Lazio, Piedmont and Veneto), where it is actively carried out (Figure 3).

In 14 regions (Basilicata, Calabria, Friuli-Venezia Giulia, Lazio, Lombardy, Molise, Piedmont, Puglia, Sicily, Umbria, Valle d’Aosta, Veneto and the autonomous provinces of Bolzano and Trento) vaccination is offered free of charge to men who have sex with men (MSM) and who request it, except for 4 regions (Calabria, Piedmont, Puglia and Veneto) where it is actively carried out (Figure 3).

Figure 1 shows the HPV vaccination offer in Italy. The age group involved in the offer and the specific type of offer are specified for each region.

Table 1 shows a summary of the vaccination offer of each region in the adolescent age (in accordance with or offered out of the NIP in co-payment) and in certain subpopulations.

All regions have an electronic register for recording the vaccination status of people undergoing the program. All regions developed an outreach campaign for the HPV primary prevention strategy by target population.

### 3.3. Secondary Prevention Strategy in the Italian Regions

All Italian regions have one or more public screening programs for cervical cancer. Screening programs usually include a PAP-test between 25 and 29 years of age and an HPV-DNA test between 30 and 64 years. If there are no positive results, a PAP-test is performed every three years, while an HPV-DNA test is every five years.

**Figure 4:**
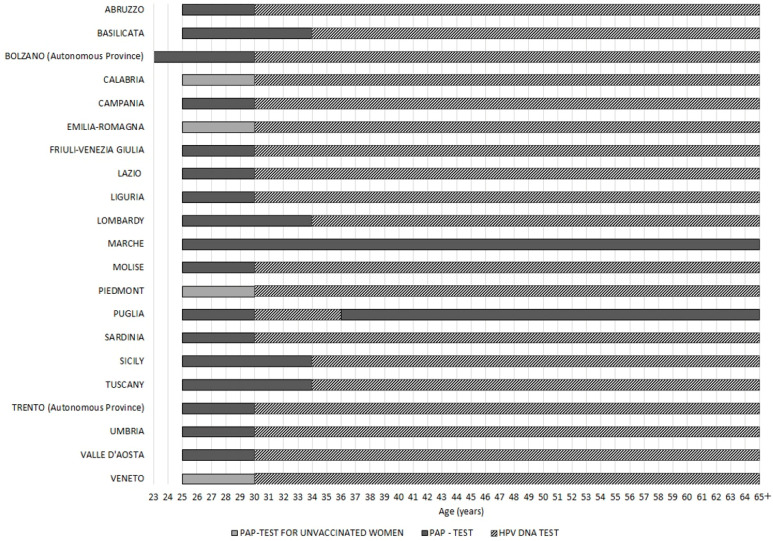
HPV secondary prevention strategy by age in Italian regions.

Marche offers a PAP-test screening every three years in women aged from 25 up to 65 years old (although this region has planned to routinely insert HPV-DNA tests starting from 1 January 2023). Instead, Puglia carries out the PAP-test screening in the age groups 25–29 and 36–64 years, while the HPV-DNA test is offered to women aged between 30 and 35 years. Four regions (Basilicata, Lombardy, Sicily and Tuscany) perform PAP-tests for women up to 33 years old and HPV-DNA tests from 34 years old onward. The autonomous province of Bolzano anticipates the beginning of the PAP-test at the age of 23 years. In four regions (Calabria, Emilia-Romagna, Piedmont and Veneto), only the women who have not been vaccinated yet are subjected to the PAP-test, while those already vaccinated begin screening at 30 years with the HPV-DNA test (Figure 4).

All regions have an electronic register for recording the screening status of people undergoing the program. Except for Emilia-Romagna, Umbria, Veneto, and the autonomous provinces of Trento and Valle d’Aosta, no other region has an integration between registers of HPV primary and secondary prevention. For some other regions (Lombardy, Marche, Veneto, the autonomous province of Bolzano and Lazio), it is a goal to be achieved in the next few years.

All regions developed an outreach campaign for the HPV secondary prevention strategy by screening type (HPV-DNA test or PAP-test).

## 4. Discussion

Elimination of cervical cancer is a global public health goal launched by the WHO in 2018 [14] as well as a target included in the Europe’s Beating Cancer Plan [16]. As a strategy for achieving this goal, the WHO emphasised the importance of cost-effective and evidence-based interventions, including vaccination of girls against HPV, screening programs, treatment of precancerous lesions, and improving access to diagnosis and treatment of invasive cancers [26]. These interventions are consistent with the Italian recommendations [10]; however, a certain inequity level can be retrieved. As a matter of fact, Italy is characterised by different health policies for the 21 regions and autonomous provinces in terms of offer and ways of implementation in prevention and treatment of HPV-related cancers and other diseases as each Italian region has, to a certain extent, its own decision-making autonomy [23].

In Italy, the Essential Levels of Assistance (LEA) are the performances, services, and benefits that the Italian National Health Service (“Servizio Sanitario Nazionale”—SSN) is required to provide to all citizens free of charge [27]. HPV-related primary and secondary prevention are both part of the LEA.

Although the common goal in Italy is to actively (by sending an invitation via letter or phone call) offer vaccination to all 11-year-old adolescents and screening for cervical cancer to 25-year-old women, policies for the other birth cohorts or subpopulations at higher risk (such as MSM (men who have sex with men), HIV+ people, people with HPV lesions at the cervix) are extremely heterogeneous across the country.

Through the review of regional institutional documents and interviews conducted with regional experts, a mapping of vaccination and screening policies in the 21 Italian regions and autonomous provinces was carried out.

Regarding HPV primary prevention, consistent with WHO directives [28], the Italian National Immunization Plan (NIP) 2017–2019 and the National Preventive Vaccination Plan PNPV 2020–2025 identify the vaccination at 11 years old as the preferable age for actively offering HPV vaccination to the entire population (females and males) [22,23]. It is also recommended to provide HPV vaccination during the first cervical screening at 25 years of age to unvaccinated women, also using the opportune occasion of the call to the first cervical screening, as a catch-up strategy, in addition to the recommendation to use the vaccination according to the guidelines of the regions (co-payment scheme) for all women. Moreover, vaccination against HPV is also recommended in other subpopulation risk groups, like MSM [23]. The new National Plan for Vaccine Prevention (PNPV) 2023–2025, which was published after the data collection for our study, stated to offer HPV vaccination starting from 11 years old for both men and women. Catch-up programs are proposed for women up to at least age 26, in addition to using it as the first screening call for cervical cancer prevention, and for men up to 18 years old. Vaccination is, in addition, recommended for women who have been treated for CIN2+ or higher grade lesions, HIV-infected individuals, and MSM. HPV vaccination is also included in the list of recommended vaccines for travellers [29].

According to our results, all Italian regions have a vaccination program for adolescents, and several regional differences exist regarding the vaccination recommendations for non-primary-target cohorts. Some regions (Basilicata, Emilia-Romagna, Veneto and the autonomous provinces of Trento and Bolzano) do not yet offer vaccination on the occasion of the call for the first PAP-test, while the other five regions (Abruzzo, Liguria, Marche, Sardinia, and Tuscany) have not foreseen an offer for MSM as of 31 December 2021. In Campania, vaccination can be anticipated from the age of 9 years, in line with the WHO recommendations, in order to anticipate sexual debut [28]. In Lombardy, the first dose of the HPV vaccine can be administered together with the second booster dose of the diphtheria-tetanus-pertussis-poliovirus vaccine or with the first dose of the quadrivalent meningococcal vaccine, as indicated by the Italian scientific societies in the last edition of the Lifetime Immunization Schedule (Calendario Vaccinale per la Vita) [30,31]. In Basilicata and Molise, HPV vaccination is offered free of charge not only to women who tested positive for the PAP-test but also to women who are positive for the HPV-DNA test.

It is, therefore, important to work to ensure that adherence to vaccination offers takes place as early as possible, to anticipate the onset of sexual activity [28]. In fact, vaccination is an economically sustainable strategy for eliminating cervical cancer and for controlling all other HPV-related cancers. It has been proven that in vaccinating 12-year-old girls (together with a screening program with intervals generally longer than one year), there is an incremental cost-effectiveness ratio (ICER) of USD 100,000 or less per QALY earned, compared to an increase in cost per QALY greater than USD 100,000 achieved through catch-up vaccination for up to 21 years [32]. In addition, vaccination with a nonavalent vaccine earns an ICER of EUR 4483 per QALY in the vaccination of female adolescents and EUR 10,463 per QALY in vaccinating boys and girls [33].

HPV vaccination in young men could improve community immunity (e.g., for unvaccinated women) and offer additional protection for MSM. A Finnish study published in 2018 showed that a gender-neutral vaccination program could improve community immunity. Other factors could be considered in the cost-effectiveness of a gender-neutral vaccination programme, such as the duration of protection offered by HPV vaccination. Furthermore, when HPV vaccine price decreases, the cost-effectiveness of universal vaccination can increase [34].

Our results showed a wide heterogeneity among regions in the offer of HPV vaccination for older birth cohorts. HPV vaccination for additional cohorts or subpopulations at higher risk represents another relevant opportunity to increase the level of protection against HPV and to reduce the clinical, epidemiological and economic burden of HPV-related diseases. As a matter of fact, there are different costs due to HPV-related diseases, which generate an important economic burden, e.g., direct costs, such as diagnostic tests, check-ups, treatments and any hospitalisations, and indirect costs, like workdays lost by patients and their families and the impact on patient quality of life [35].

From an economic point of view, the savings obtainable in the case of extension of the HPV vaccination to patients treated for a previous HPV-related cervical lesion were also calculated. This extension would entail a lower expenditure for the SSN equal to EUR 155,596.38 in 5 years due to the lower incidence of HPV-related lesions. Few studies have shown a reduction in premature births due to HPV lesions; however, this association needs to be explored in future studies [36,37].

Moreover, Deshmukh et al., through cost-effectiveness modelling, concluded that post-treatment quadrivalent HPV vaccination for MSM living with HIV infection aged 27 years and older is cost-effective in Italy [38], and, in addition, HPV vaccination has been shown to be safe and immunogenic in HIV-positive MSM [39]. In this regard, from our analysis, it appears that more than half of the Italian regions offer free HPV vaccination to MSM and people living with HIV, and it would be essential to implement it throughout the national territory.

Regarding HPV secondary prevention, LEA provides [40] an active call and execution of first and second-level screening tests to the target population.

In particular, in Italy, the 12 high-risk types (HPV 16, 18, 31, 33, 35, 39, 45, 51, 52, 56, 58, 59), plus 1, 2 or 3 probable/possible high-risk types (HPV 66, 67, 68) are detected by screening [41].

It is certainly necessary to activate programs and coordinate actions in order to actively encourage the population to adhere to the opportunities provided by the previous national prevention plan [42]. In addition, in the PNP 2014–2018, the launch of the screening program for cervical cancer introducing the HPV-DNA test was foreseen within 2018 [43]. According to our results, this goal was not achieved in all regions. For example, in the Marche Region, the introduction of the HPV-DNA test is expected to be introduced in 2023.

The current Italian PNP 2020–2025 claims to continue the consolidation of organised screening programs for cancer prevention with the aim to complete the transition to the model based on the primary HPV-DNA test for cervical cancer screening. The PNP also requires adapting the screening campaign to the new vaccination situation, considering that in the two-year period 2021–2022, girls vaccinated against HPV in the 12th year of life have reached the age of access to the first screening test. Although cervical HPV screening has few undesirable effects and the treatment of pre-invasive lesions is particularly safe, this could involve an increase in preterm births. Therefore, it is important to evaluate the different epidemiology of HPV and cervical lesions in the vaccinated population compared to the unvaccinated one in order not to expose young women to an overdiagnosis and overtreatment risk [22,44]. According to our results, only three regions (Emilia-Romagna, Piedmont and Veneto) distinguish between vaccinated and unvaccinated girls at the time of screening.

Organized cancer screening programs are not homogeneous throughout the national territory. From the Italian PASSI Survey 2020–2021 (the PASSI, Progress of Health Authorities for Health in Italy, is a surveillance system that continually investigates various aspects of health status, lifestyle habits, as well as the provision and utilisation of prevention programs and road and home safety), it appears that in Italy, 77% of women between 25 and 64 years of age undergo cervical screening (PAP-test or HPV-DNA test), with a clear north–south gradient: coverage is, on average, equal to 85% in the northern and central Italy regions (91% in the autonomous province of Bolzano) and 69% in the southern regions (with minimum coverage for some regions, such as Molise, 63%, or Campania and Calabria, 65%). As observed for vaccinations, in 2020, there was a drop in the coverage for cervical screening, which recovered in 2021, but without returning to pre-pandemic values. Over time, there has been a statistically significant increase in the female population, who adhere to organised screening programmes, and a decrease in spontaneous screening [45].

Self-sampling is a possible method to implement screening coverage among women: it is already done in some countries of the world, such as Sweden as part of the cervical cancer elimination strategy, while in Italy, it is done only experimentally in some settings and not systematically. In Sweden, in particular, self-sampling is a method that saw its first use during the pandemic as an emergency screening method and, given its success, has been implemented routinely since 2022 [46]. Clearly, it is important that self-sampling be embedded within an organised screening program to be effective and to ensure that every woman is tested at the right time [47].

PNP 2020–2025 recommends integration of the systems for recording data on the vaccination status and on the adhesion to HPV screening tests [22] in order to be able to compare them and monitor the effectiveness of preventive interventions. Actually, our results showed that most regions do not have an integrated register (with the exception of Emilia-Romagna, Umbria, Veneto, Autonomous Province of Trento and Valle d’Aosta), but in some regions, including Lazio, Lombardy, Marche, Autonomous Province of Bolzano, it is expected to be introduced shortly.

Lastly, the new PNPV 2023–2025 includes among its objectives that of strengthening the prevention of cervical cancer and other HPV-related diseases, an objective that can certainly also be achieved through greater homogenisation of prevention strategies at a national level.

This study has some limitations. First, the response rate of the regional experts enrolled in the validation phase was less than 100%. The data were not homogeneous because, in some cases, updates to 2022 were provided by the regional experts. When the validation was not completed, the data were updated to 31 December 2021. Moreover, it was not always possible to verify the correspondence between what was issued in the regional official documents, what was reported by the experts who validated the data and what was implemented in practice in the regions. The assessment of the results depends on the interpretation of the official regional documents by the authors, the specific skills of the involved experts and the information gathered. The study involved the analysis of numerous data from different sources and communication channels, creating a possible risk of fragmentation of information. On the other hand, to the best of our knowledge, it is one of the first Italian analyses to offer an updated and comprehensive overview of the state-of-the-art of primary and secondary HPV prevention.

## 5. Conclusions

The analysis of official regional documents, online platforms or institutional websites shows that the HPV prevention programmes of most Italian regions follow the most up-to-date national indications of the Italian health authorities. On the other hand, there are evident differences between the regions in the application of the HPV preventive strategies. Therefore, it is necessary to foresee actions of coordination that can integrate, constantly update and share in real-time reliable and correct information on the offer of HPV primary and secondary prevention.

In conclusion, it is crucial to establish innovative models that aim to harmonise the preventive offer at the national level and guarantee equity in the access to prevention programmes. The data we collected could enrich evidence on the impact of public health recommendations and drive future prevention strategies. Moreover, outcomes provided by such studies could properly inform the regional and national public health decision-makers and strengthen the resiliency of preventative programmes. In this view, useful actions are (1) the implementation of informatic recording systems, (2) the integration of the primary and secondary prevention activities, in such a way as to have a constantly updated overview of vaccination coverage and screening for all target populations at a national level, and the improvement in training for healthcare professionals and health promotion initiatives towards the general population. Only in this way could it be possible to increase the adherence and the offer of vaccination and screening with the final goal of reaching HPV disease elimination.

## Figures and Tables

**Table 1:** Summary of the HPV vaccination offered in adolescence cohorts and in certain subpopulations. F = female; M = male.

Region	Free of Charge Offer for Cohorts According to NIP		Free of Charge Offer for Subpopulation			Co-Payment for Additional Cohorts Compared to NIP
	At 11 Years Old	Without Age Limit	HIV+	MSM	HPV Genital Warts	
Abruzzo	F; M					
Basilicata	F; M		F; M	M	F	F
Calabria	F; M		F; M	M	F	F
Campania	F; M				F	
Emilia-Romagna	F; M		F; M		F	F; M
Friuli-Venezia Giulia	F; M		F; M	M	F	F; M
Lazio	F; M		F; M	M	F	F; M
Liguria	F; M				F	F; M
Lombardy	F; M		F; M	M	F	F; M
Marche	F; M		F; M		F	
Molise	F; M			M	F	F; M
Piedmont	F; M	F; M	F; M	M	F	F; M
Puglia	F; M			M	F	F; M
Sardinia	F; M				F	F
Sicily	F; M		F; M	M	F	F; M
Tuscany	F; M				F	F; M
Trentino-Alto Adige—Autonomous Province of Trento	F; M		F; M	M	F	F
Trentino-Alto Adige—Autonomous Province of Bolzano	F; M			M	F	F; M
Umbria	F; M			M	F	
Valle d’Aosta	F; M		F; M	M	F	F
Veneto	F; M		F; M	M	F	F; M

## Data Availability

The data were collected in the form of an interview and reported in the manuscript.
